# The livelihoods of Haitian health-care providers after the january 2010 earthquake: a pilot study of the economic and quality-of-life impact of emergency relief

**DOI:** 10.1186/1865-1380-5-13

**Published:** 2012-03-02

**Authors:** Rohini J Haar, Sassan Naderi, John R Acerra, Maxwell Mathias, Kumar Alagappan

**Affiliations:** 1 North Shore University Hospital/Long Island Jewish Medical Center, Department of Emergency Medicine, 300 Community Drive, Manhasset, NY 11030, USA

## Abstract

**Introduction:**

An effective international response to a disaster requires cooperation and coordination with the existing infrastructure. In some cases, however, international relief efforts can compete with the local work force and affect the balance of health-care systems already in place. This study seeks to evaluate the impact of the international humanitarian response to the 12 January 2010 earthquake on Haitian health-care providers (HHP).

**Methods:**

Fifty-nine HHPs were surveyed in August of 2010 using a modified World Health Organization Quality of Life-Brief questionnaire (WHOQoL-B) that included questions on respondents' workload before the earthquake, immediately after, and presently. The study population consisted of physicians, nurses, and technicians at public hospitals, non-governmental organization (NGO) clinics, and private offices in Port-au-Prince, Haiti.

**Results:**

Following the earthquake, public hospital and NGO providers reported a significant increase in their workload (15 of 17 and 22 of 26 respondents, respectively). Conversely, 12 of 16 private providers reported a significant decrease in workload (*p *< 0.0001). Although all groups reported working a similar number of hours prior to the earthquake (average 40 h/week), they reported working significantly different amounts following the earthquake. Public hospital and NGO providers averaged more than 50 h/week, and private providers averaged just over 33 h/week of employment (*p *< 0.001).

Health-care providers working at public hospitals and NGOs, however, had significantly lower scores on the WHOQoL-B when answering questions about their environment (*p *< 0.001), and in open-ended responses often commented about the lack of potable water and poor access to toilets. Providers from all groups expressed dissatisfaction with the scope and quality of care provided at public hospitals and NGO clinics, as well as disappointment with the reduction in patient volume at private practices.

**Conclusions:**

The emergency medical response to the January 2010 earthquake in Haiti had the unintended consequence of poorly distributing work among HHPs. To create a robust health-care system in the long term while meeting short-term needs, humanitarian responses should seek to better integrate existing systems and involve local providers in the design and implementation of an emergency program.

## Introduction

Humanitarian organizations may have both positive and negative impacts on the socioeconomic state of a post-disaster community [1-4]. Acutely, humanitarian relief can provide the necessary resources to moderate the destructive effects of a natural or man-made disaster. However, in protracted emergencies, foreign aid has both beneficial and detrimental consequences [5]. Socioeconomic studies on developing countries detail the effects of humanitarian aid on resources such as food, specifically stating that prolonged humanitarian support can negatively impact the livelihoods of local farmers and business people [5,6]. There may be a similar impact on the local health economy by creating redundant health systems that compete with local providers [7,8]. One issue yet to be studied is the effect of emergency humanitarian aid on the existing local health-care providers. The transition between emergency response and long-term program development provides a crucial opportunity to observe the effects of aid on the local health-care system and, in the future, to improve the systems of disaster-affected people both during and after a crisis. In particular, evaluating the role of local service providers within this transition provides a more nuanced understanding of health systems, local economics, and livelihoods affected by the aid industry.

Despite years of slow progress, pre-earthquake Haiti had a health system inadequate to treat the patients suffering from chronic conditions [9,10]. The earthquake in Haiti on 12 January 2010 displaced populations and increased the health requirements of an already overwhelmed system [11]. Since the earthquake, there has been an enormous response from the international community in the form of equipment and pharmaceutical donations, field hospitals, and numerous internationally trained medical staff [12-14]. The restructuring of the health system to include these new providers raises questions about the effect of aid on local health-care providers [15,16]. This study surveys the perceived effect of NGOs and other humanitarian aid on the quality of life and workload of the local physicians, nurses, and health-care technicians of Port-au-Prince, Haiti.

## Methods

Fifty-nine Haitian health-care providers (HHP) were surveyed in August 2010 using a modified World Health Organization Quality of Life-Brief questionnaire (WHOQoL-B) [17,18]. The WHOQoL-B is a 26-item validated survey tool that divides quality of life into four domains: physical, psychological, social, and environmental [19-21]. Written surveys were available in English, French, and Haitian Créole, though all respondents chose the French version. An interpreter was available for translation between English, French, and Haitian Créole, and to answer any questions upon request. The WHOQoL has been validated in numerous countries but not yet in Haiti. This may affect the full ability to analyze changes in quality of life from the baseline in this nation; however, without any sourceable, validated, quality-of-life surveys in Haiti, the WHOQoL was the most widely validated and appropriate survey tool. Furthermore, as this survey seeks to compare perceived changes between pre-earthquake and post-earthquake quality of life in each respondent, the authors hope this limits the bias from using a survey that is not validated in this specific country. Questions were added to include information on health-care providers' area of work, hours, and quantity of work, satisfaction with services pre- and post-earthquake, and ability to find employment post-earthquake (see Appendix). The study population consisted of physicians, nurses, and other skilled clinical hospital workers such as nursing assistants and surgical technicians. They were identified by visiting hospitals, non-governmental organization (NGO) clinics, and private offices in Port-au-Prince. Respondents were categorized as public (working at a government-funded hospital, specifically the Hôpital Université d'Etat d'Haiti (known as HUEH), private (a privately run medical practice, either hospital, clinic, or office, that charges patients for care), or non-governmental providers (any hospital or clinic that is funded and organized by a known international NGO functioning in Haiti). Once completed, the numerical data were compiled using statistical software, and the short-answer data were translated from French into English by RH and MM.

Internally displaced person (IDP) camp sites with medical clinics and local health facilities in Port-au-Prince were identified by lists provided by NGOs, local contacts, or on the health cluster website http://www.oneresponse.info. Surveys were distributed to providers in free clinics and private providers in the surrounding area for comparative data. Surveys were also conducted in other areas without nearby IDP camps to compare a broader range of private providers. Though this is a convenience sample, the authors attempted to identify equal numbers of clinical staff from all three groups. Physicians, nurses, and other clinical health-care staff were all surveyed to assess the effects on all skilled clinical health-care workers in the area despite their vastly different skill sets, and because different medical facilities had different proportions of physicians, nurses, and technicians in accordance with the acuity of their patients. As this study hoped to examine the effects of humanitarian assistance from numerous clinical realms and the potential displacement of local staff, it was necessary to survey different types of clinical workers in a more comprehensive picture of the health-care sector.

This study was approved by the Institutional Review Board of North Shore University Hospital. At the time of the study, there was no research review institution in Haiti from which to request approval. However, permission was taken from administrators at all hospitals and clinics surveyed.

## Results

**Figure 1:**
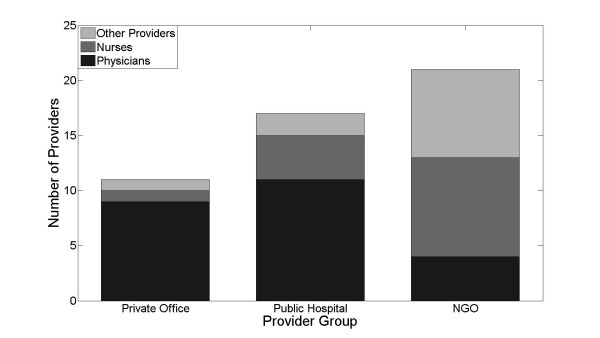
**Most respondents from private offices were physicians, while respondents from public hospitals and NGOs included a large number of nurses and other types of health-care providers**.

Eighty-eight HHPs were approached with the survey, and 59 (67%) agreed to participate. Of the 29 providers that did not respond, 7 were private providers (6 physicians, 1 nurse), 17 were public sector (9 physicians, 7 nurses, and 1 nursing assistant), and 5 were from the public sector (2 physicians, 3 nurses). Of the 59 health-care providers that were surveyed, 16 were private health-care providers, 17 were public health-care providers, and 26 were employed by non-governmental organizations (NGOs). Among all providers, there were 32 physicians, 20 nurses, and 7 nursing assistants and other technical staff (Figure 1).

**Figure 2:**
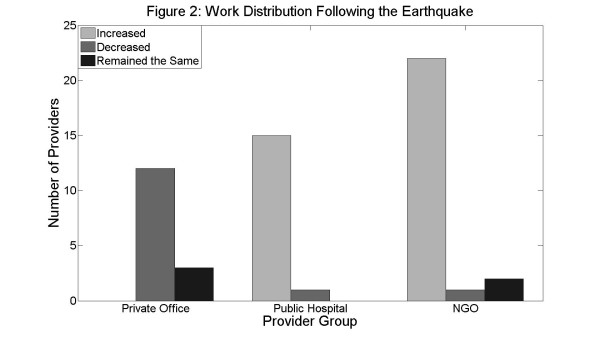
**Most of the respondents who worked in private offices reported a decrease in their workload following the earthquake, while respondents from public hospitals and NGOs reported an increase**.

Providers at public hospitals and NGOs stated they had a substantial increase in workload (15/17 and 22/26 respondents, respectively). Conversely, 12/16 private providers stated they saw a significant decrease in workload (*p *< 0.0001; Figure 2).

**Figure 3:**
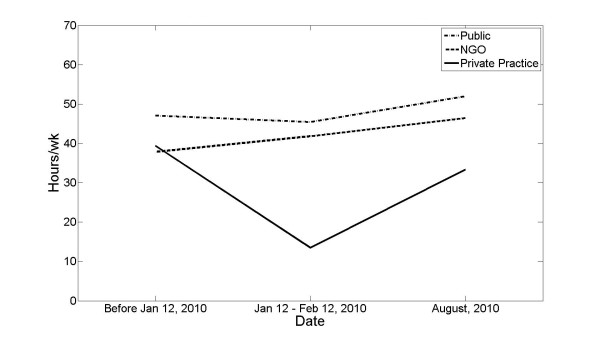
**A graph showing the number of hours worked before the earthquake, immediately after the earthquake, and at the time of the survey demonstrates how the distribution of work shifted away from private health-care providers and toward public hospitals and NGOs**.

Although all groups were similar before the earthquake in the number of hours they reported working (total average 40 h/week), they differed significantly both immediately after the earthquake and 8 months later. NGO and public providers reported that between 12 February and 12 March, their workload was sustained at nearly 45 h/week. In the same time range, private providers had a considerable decrease in workload to 13 h/week. By August 2010, this significant difference still had not normalized. Public and NGO providers worked 52 and 46 h/week, respectively, while private providers worked just over 33 h/week of employment (*p *< 0.001; Figure 3).

**Figure 4:**
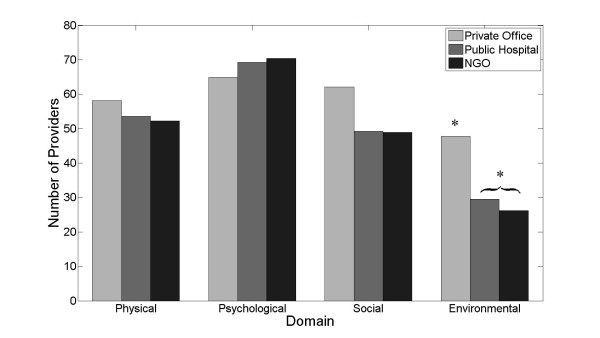
**Survey responses were scored using the WHOQoL-Bref protocol **[17]. Health-care providers working at public hospitals and NGOs reported significantly worse conditions when answering questions about their work and home environment (*p > 0.05).

In the quality-of-life responses, health-care providers working at NGOs and public hospitals had significantly lower scores on the WHOQoL-B when answering questions within the environment domain (26.2 and 29.5 out of 100, respectively, vs. 47.8 for private providers; *p *< 0.001; Figure 4). There were no significant differences found between NGO and public providers. Environmental domain questions focus on financial resources, physical safety and security, transportation, social accessibility, home environment, physical environment, and opportunities for leisure and education [17-20,22]. Open-ended responses within the modified survey indicate that the concerns of the groups were different: private providers expressed frustration with lack of patient volumes and support in rebuilding offices; public provider respondents discussed concern over future jobs and poor working conditions; NGO employees expressed unease with cleanliness, poor transportation, and the security of their jobs if international funding decreased. These open-ended questions also allowed for private health-care providers to communicate their opinions as to why their patient volumes have dropped, including the death of patients, migration or displacement away from the site of the medical care, confusion with new office locations, and worsening poverty (Table 1).

**Table 1:** Respondents from the three groups expressed both positive and negative sentiments regarding their lives following the earthquake.

Public hospital employees	Private providers	NGO employees
Negative comments		
Lack of cleanliness. Lack of medications. Lack of respect from the administration. Inconsistent laboratory tests. The increase in work hours without a cafeteria	NGOs don't take good care of patients so patients end up getting referred to me in worse condition	Sometimes the noise. A lot of water on the ground when it rains
The heat. Sometimes the noise of the generator, the lack of water	Not a lot of private jobs. My clinic closed, so now I have to work at another clinic	The roofing is canvas
1) Lack of specialists and education of the residents 2) Unavailable medications 3) Defective medical testing 4) Late salary payments	No jobs, young doctors that are not fully trained and qualified are taking NGO jobs	The premises are poorly structured, which is risky for those of use who take care of the sick. There's no privacy to consult with patients
There hasn't been any change in the conditions. If anything, it's gotten worse	NGOs don't treat patients properly so patients are very unsatisfied and return to my office. NGOs provide expired medications	The intense heat under the tent. The lack of interest of the patients to come consult a doctor
Just after the earthquake there was a lot of improvement, but now we are returning to where we started	I have an office but no patients. A lot of patients died or moved away. A lot don't have any money and can't pay for care. Patients don't know where I am	No bathrooms for the health-care workers. Long days exposed to the sun. No chairs in the waiting room. No means of transportation for displaced workers. No drinkable water for the workers
Lack of water. Lack of cleanliness, no cafeteria	In general, many in this clinic are complaining that it is empty. I have an 80% patient reduction	
**Positive comments**		
We now have water to drink. The number of consultations has decreased, but we have more specialist consultations like family planning, OB/GYN	I own my own office so I have more control	The patients are understanding and collaborate with the doctors
In terms of medical practice, I'm getting a solid experience with the number and variety of cases I see	I maintain clean working conditions	Despite the negative aspects, I have the opportunity to help the patients medically. We have access to things like the Malaria tests, blood sugar levels, bandages, urine tests, etc.
The teaching restarted and we're no longer in tents with unbearable heat. We can take care of our patients according to our protocols. There are a lot more medications and supplies to take care of patients then previously	I own my practice and my time	There are a lot of people who found jobs
Devotion of residents to provide care. Collaboration is more or less perfect. We help each other	NGOs are providing some employment for those without enough work	We are receiving lessons on psychiatric evaluation which is positive

## Discussion

The results of the survey provide compelling evidence showing that there is a significant difference in quality of life and perceived livelihoods among private, public, and NGO-employed health-care providers in Haiti before the after the January 2010 earthquake and the humanitarian response that followed. Our study indicates that despite the increased health-care needs of the Haitian population and increased patient volumes at public hospitals and NGO clinics, the private health-care providers in urban Port-au-Prince have experienced a reduction in patient volume that has impacted their livelihoods. Furthermore, while the NGOs and public providers have a comparative increase in workload, they report working in unhealthy and unsafe environments.

The relative similarity in work volumes between public hospital and NGO providers indicates the necessity of quality health care at low cost among Haitian patients. NGO-sector providers expressed concern that work volume is changing with the population of the camps they are located in, and that international support and interest for their work seems to be decreasing despite the large populations that remain in camps.

Further, the quality-of-life data indicate that health care in public and NGO sectors may be taking place in less sound, more unhygienic environments than in the private sector. Despite the similarity in the responses to the social, psychological, and physical domains of the survey, there is a significant difference in the results of the environmental domain. Qualitative responses elucidate potential causes of this difference. NGO and public sector providers made statements concerning long transport times to sites, poor hygiene in the clinical areas, lack of washrooms and running water, lack of privacy when examining patients, and, in NGOs in particular, a lack of job security. The similarities in the social, psychological, and physical domains are supported by qualitative data showing that the quality of life of HHPs is affected by the same issues of health, personal relationships, social support, and spirituality, irrespective of workplace.

Qualitative survey responses indicate a number of possibilities as to why private health-care providers have continued to have a perceived decreased workload despite the presumably increased health-care needs of the population. Factors mentioned in open-ended questions include the death of many patients, migration or displacement away from the site of the medical care, and confusion with new office locations or hours if providers lost their offices in the earthquake or did not open their offices immediately after the earthquake. Respondents from all groups also reported a loss of social structure, community, communications, and monetary savings as well as worsening poverty that contributed to the inability of patients to afford care or unwillingness to pay when free care is readily available.

### Limitations

There are several limitations of this study. Because of the continued upheaval in Port-au-Prince geographically, politically, and within populations, it was difficult to identify providers who were still working. Both within camps and among private providers, the day-to-day situation was not consistent, thereby limiting our study to accessible and available providers. This methodology may have missed unemployed or underemployed providers as well as those in more remote locations. Because of the difficulty of traversing poor roads, some inaccessible offices and hospitals may not have been reached. Occupation, specifically among physicians in Haiti, is complicated in that the same physician may work in a public hospital some days and privately in his/her own office other days. These factors may have biased the results in different ways that would require further investigation. Because the WHOQoL was not specifically validated in Haiti prior to this study, the results of the survey can only compare the respondents' quality of life before and after the earthquake and not to quality-of-life data generally. As this study was a convenience sample-based survey, the results must be considered an initial observation of possible problems and concerns rather than a definitive conclusion about the effect of humanitarian aid on local health-care workers. Finally, since the sample was small and difficult to access, a study with more resources could more specifically sub-group different types of clinicians such as physicians, nurses, and other staff into separate groups to understand quality-of-life changes both between and among these different groups. The limitations enumerated here should be accounted for in a more thorough follow-up evaluation.

## Conclusions

There are many patients in Haiti requiring quality medical care, but the uneven distribution of work and the discontent among local health-care workers, in part created by a new health system that is functioning in parallel with the previous system, have added to the upheaval in the Haitian health-care sector. Our study, though a broad observational one, indicates that the livelihoods of local health workers have likely been negatively affected not only by the tremendous deleterious impact of the earthquake itself, but also, months later, by the humanitarian aid organizations that sought to alleviate its effects. The quality-of-life data that indicate that the decrease in workload witnessed immediately after the earthquake during the height of the humanitarian response was only partly eased months later, possibly because the transition from a fully humanitarian aid response to one that integrated the local health-care system was not successful yet. Because local providers are the backbone of the health-care sector, it is vital for all actors, governmental and non-governmental, to create active dialogue on this issue. There will certainly be difficulty in accessing and motivating private providers to tackle the management of impoverished communities that may not be able to pay sufficiently, but creative solutions such as subsidizing care for poor people within the private system have been utilized in some situations and may prove useful in Haiti [7,23,24]. As Haiti rebuilds over the next several years, local, governmental and international organizations must leverage the enormous potential of the providers already present to more equitably distribute work and create a more effective health-care infrastructure.

## Competing interests

The authors declare that they have no competing interests.

## Authors' contributions

RJH administered and collected the surveys in Haiti. All authors contributed equally to the design of the study and the development of the manuscript. All authors read and approved the final manuscript.
